# Accuracy of Manual Intracranial Pressure Recording Compared to a Computerized High-Resolution System: A CENTER-TBI Analysis

**DOI:** 10.1007/s12028-023-01697-2

**Published:** 2023-03-15

**Authors:** Tommaso Zoerle, Tatiana Birg, Marco Carbonara, Peter Smielewski, Michal M. Placek, Elisa R. Zanier, Cecilia A. I. Åkerlund, Fabrizio Ortolano, Nino Stocchetti, CENTER-TBI High Resolution ICU Sub-Study Participants and Investigators, Audny Anke, Ronny Beer, Bo-Michael Bellander, Erta Beqiri, Andras Buki, Manuel Cabeleira, Arturo Chieregato, Giuseppe Citerio, Hans Clusmann, Endre Czeiter, Marek Czosnyka, Bart Depreitere, Ari Ercole, Shirin Frisvold, Raimund Helbok, Stefan Jankowski, Daniel Kondziella, Lars-Owe Koskinen, Ana Kowark, David K. Menon, Geert Meyfroidt, Kirsten Moeller, David Nelson, Anna Piippo-Karjalainen, Andreea Radoi, Arminas Ragauskas, Rahul Raj, Jonathan Rhodes, Saulius Rocka, Rolf Rossaint, Juan Sahuquillo, Oliver Sakowitz, Peter Smielewski, Nina Sundström, Riikka Takala, Tomas Tamosuitis, Olli Tenovuo, Andreas Unterberg, Peter Vajkoczy, Alessia Vargiolu, Rimantas Vilcinis, Stefan Wolf, Alexander Younsi, Frederick A. Zeiler

**Affiliations:** 1 Neuroscience Intensive Care Unit, Department of Anesthesia and Critical Care, Fondazione IRCCS Ca’ Granda Ospedale Maggiore Policlinico, Milan, Italy; 2 Department of Pathophysiology and Transplantation, University of Milan, Milan, Italy; 3 Brain Physics Lab, Division of Neurosurgery, Department of Clinical Neurosciences, Addenbrooke’s Hospital, University of Cambridge, Cambridge, UK; 4 Department of Neuroscience, Istituto di Ricerche Farmacologiche Mario Negri IRCCS, Milan, Italy; 5 Department of Physiology and Pharmacology, Section of Perioperative Medicine and Intensive Care, Karolinska Institutet, Stockholm, Sweden

**Keywords:** Traumatic brain injury, Intracranial pressure, Cerebral perfusion pressure, Data collection

## Abstract

**Background:**

Monitoring intracranial pressure (ICP) and cerebral perfusion pressure (CPP) is crucial in the management of the patient with severe traumatic brain injury (TBI). In several institutions ICP and CPP are summarized hourly and entered manually on bedside charts; these data have been used in large observational and interventional trials. However, ICP and CPP may change rapidly and frequently, so data recorded in medical charts might underestimate actual ICP and CPP shifts. The aim of this study was to evaluate the accuracy of manual data annotation for proper capturing of ICP and CPP. For this aim, we (1) compared end-hour ICP and CPP values manually recorded (MR) with values recorded continuously by computerized high-resolution (HR) systems and (2) analyzed whether MR ICP and MR CPP are reliable indicators of the burden of intracranial hypertension and low CPP.

**Methods:**

One hundred patients were included. First, we compared the MR data with the values stored in the computerized system during the first 7 days after admission. For this point-to-point analysis, we calculated the difference between end-hour MR and HR ICP and CPP. Then we analyzed the burden of high ICP (> 20 mm Hg) and low CPP (< 60 mm Hg) measured by the computerized system, in which continuous data were stored, compared with the pressure–time dose based on end-hour measurements.

**Results:**

The mean difference between MR and HR end-hour values was 0.02 mm Hg for ICP (SD 3.86 mm Hg) and 1.54 mm Hg for CPP (SD 8.81 mm Hg). ICP > 20 mm Hg and CPP < 60 mm Hg were not detected by MR in 1.6% and 5.8% of synchronized measurements, respectively. Analysis of the pathological ICP and CPP throughout the recording, however, indicated that calculations based on manual recording seriously underestimated the ICP and CPP burden (in 42% and 28% of patients, respectively).

**Conclusions:**

Manual entries fairly represent end-hour HR ICP and CPP. However, compared with a computerized system, they may prove inadequate, with a serious risk of underestimation of the ICP and CPP burden.

**Supplementary Information:**

The online version contains supplementary material available at 10.1007/s12028-023-01697-2.

## Introduction

Intracranial pressure (ICP) monitoring is indicated in patients with severe traumatic brain injury (TBI) admitted to intensive care units (ICUs) [1]. It is fundamental in order to detect intracranial hypertension and/or low cerebral perfusion pressure (CPP) to establish appropriate treatments and potentially improve patient outcome [2, 3]. ICP and CPP data in the last few decades have served to explore the impact of these parameters on TBI outcome [4–9] and the efficacy of specific treatments and management protocols in observational studies and clinical trials [11–15].

The technologies and methods employed to acquire, store, and analyze ICP and CPP data in the literature vary, depending on the aims of the researchers and on the resources available [16]. High-resolution (HR) systems (sampling at frequencies higher than 100 Hz) allow continuous accurate data accumulation but are expensive and require trained staff. As a consequence, manual hourly recording of ICP and CPP on bedside charts or in case report forms (CRFs) is more common in clinical practice, and despite possible limitations, manual systems have been used in important multicenter studies [12–14, 17]. However, retrospective analysis from small single-center cohorts of patients [18, 19] indicate that these intermittent ICP and CPP values under-represent the severity and instability of intracranial hypertension.

We hypothesized that intermittent recording of ICP and CPP might underestimate the occurrence and intensity of high ICP and low CPP. The main objectives of our study were the following:

1. To compare, in a large multicenter cohort of patients, manually recorded (MR) end-hour ICP and CPP with ICP and CPP recorded simultaneously by a computerized HR system
2. To analyze whether intermittent MR ICP and MR CPP are reliable indicators of the burden of intracranial hypertension and low CPP.

## Methods

### Patients

**Fig. 1:**
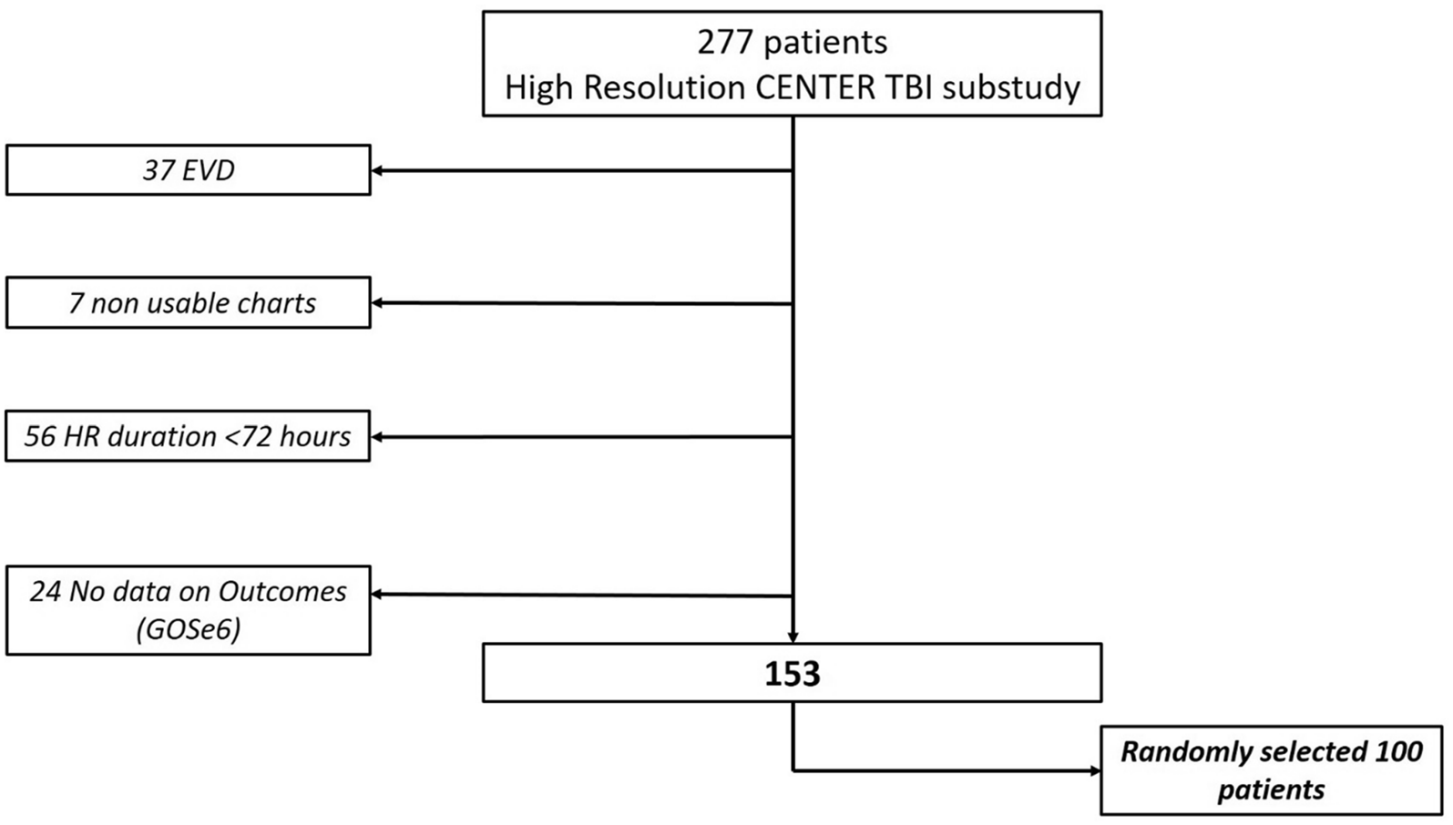
Flowchart for patients inclusion

Of 2,138 ICU patients enrolled in the Collaborative European Neuro Trauma Effectiveness Research in TBI (CENTER-TBI) trial (NCT02210221, registered on August 6, 2014), a subgroup of 277 patients had high-frequency digital signals from ICU monitoring (full waveform resolution at sampling frequencies of at least 100 Hz, provided by bedside monitors connected to dedicated computerized systems), and this was referred to as the High-Resolution CENTER-TBI Sub-Study (HR CENTER-TBI). These patients were enrolled in 21 centers from January 2015 to December 2017 and were treated in accordance with current evidence-based guidelines for TBI. All patients from this cohort were considered for this study. We further selected only patients with HR ICP monitoring lasting more than 72 h and with long-term functional outcome data (i.e., 6-month Glasgow Outcome Scale Extended score). Patients with external-ventricular-drain-based ICP data were excluded given the interruptions of ICP recording for cerebrospinal fluid drainage. In all, 100 patients were randomly selected (Fig. 1).

Data collection in the CENTER-TBI study adhered to ethical standards; medical ethics committees of all participating centers approved the study. Informed consent was obtained in accordance with local regulations. The list of sites, ethical committees, approval numbers, and approval dates can be found on the website https://www.center-tbi.eu/project/ethical-approval.

### Data Collection

As part of recruitment to the HR CENTER-TBI Sub-Study, all patients had demographic, injury, and imaging data prospectively recorded using a Web-based electronic CRF (Quesgen e-CRF, Quesgen Systems Inc, hosted on the International Neuroinformatics Coordinating Facility (INCF) platform and extracted via the INCF Neurobot tool, INCF, Sweden). For this study, basic admission demographics and centrally reported computed tomography (CT) variables for each patient’s first available CT scan were extracted using a bespoke data management tool (Neurobot [http://neurobot.incf.org] data version 2.1).

### MR ICP and CPP

End-hour ICP, systolic blood pressure (SBP), and diastolic blood pressure (DBP) were MR in the CRF every 2 h. Mean arterial pressure (MAP) was calculated as (SBP + 2 × [DBP])/3, and CPP was calculated as MAP − ICP.

### HR ICP and CPP

Data were collected using ICM + software (Cambridge Enterprise Ltd., Cambridge, UK, http://icmplus.neurosurg.cam.ac.uk) or the Moberg CNS monitor (Moberg Research Inc, Ambler, PA, USA, https://www.moberg.com), or both. MAP was obtained through arterial lines connected to pressure transducers. ICP was acquired from an intraparenchymal strain gauge probe (Codman ICP MicroSensor; Codman & Shurtleff Inc., Raynham, MA) or a parenchymal fiber optic pressure sensor (Camino ICP Monitor, Integra Life Sciences, Plainsboro, NJ; https://www.integralife.com/). CPP was calculated as specified above. The whole process of HR CENTER-TBI signal acquisition and data processing is described in previous publications [9].

### End-Hour Analysis

**Fig. 2:**
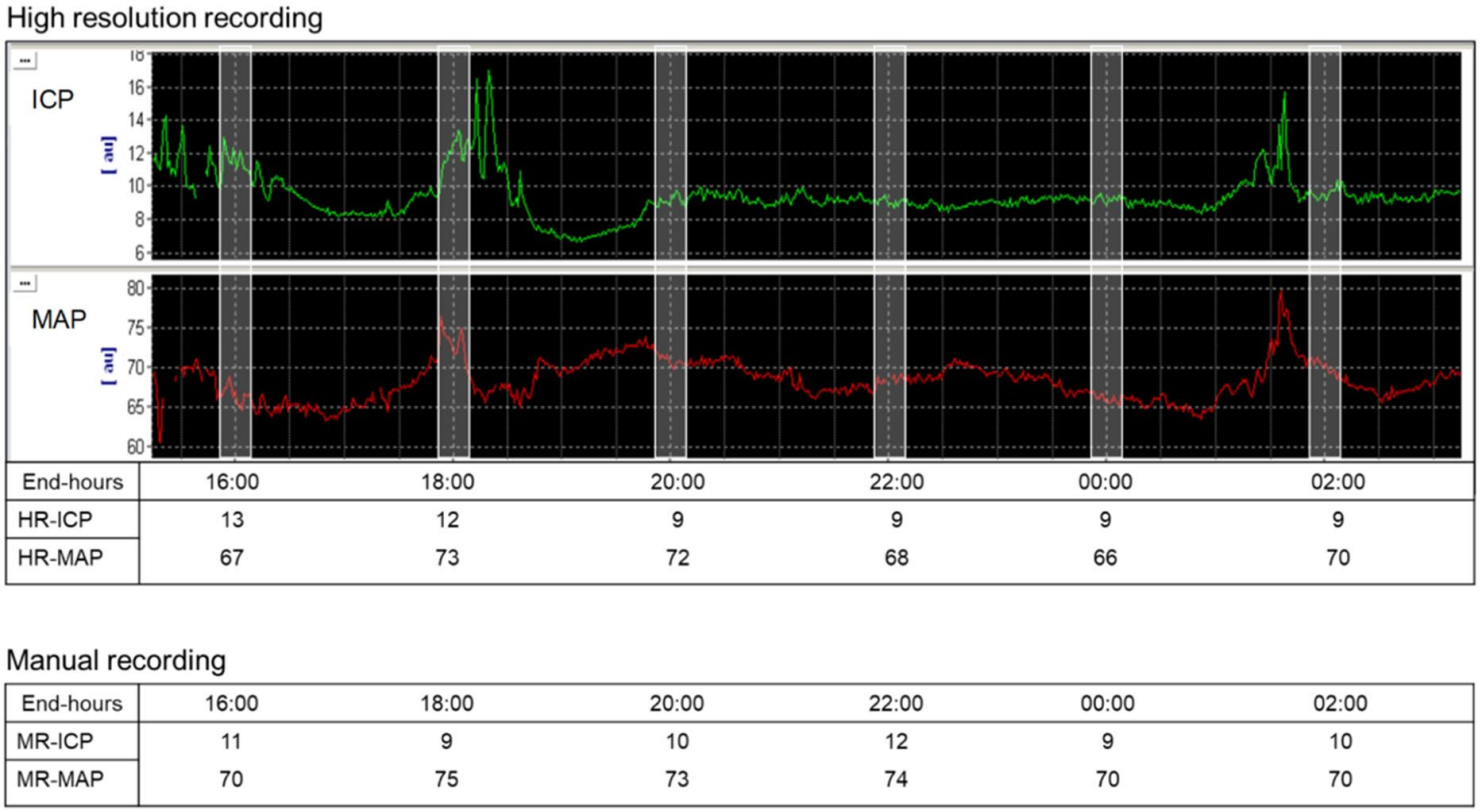
End-hour high-resolution (HR) and manual intracranial pressure (ICP) and mean arterial pressure (MAP) recordings. HR recording (upper panel): for every patient, we collected the end-hour ICP and MAP (considered as average of the ten minutes over the o’clock) corresponding to the manually recorded (MR) values. CPP was calculated as MAP − ICP. MR (lower panel): for every patient, we extracted from the case report form (CRF) the end-hour ICP and MAP entered every 2 h. MAP was calculated as (SBP + 2 × [DBP])/3, and CPP was calculated as MAP − ICP

For comparison we identified the end-hour ICP and CPP in the HR recording (considered as average of the ten minutes over the o’clock) corresponding to the MR values entered in the CRF (Fig. 2).

To check the match between different recordings we analyzed the following: (1) MR values (both ICP and CPP) and the corresponding end-hour HR values, (2) the number of MR and end-hour HR ICP > 20 mm Hg and the number of MR and end-hour HR CPP < 60 mm Hg, (3) the number of MR and end-hour HR ICP with a difference of more than 5 mm Hg and the number of MR and end-hour HR CPP with a difference of more than 10 mm Hg.

### ICP and CPP Burden (Pressure–Time Dose)

**Fig. 3:**
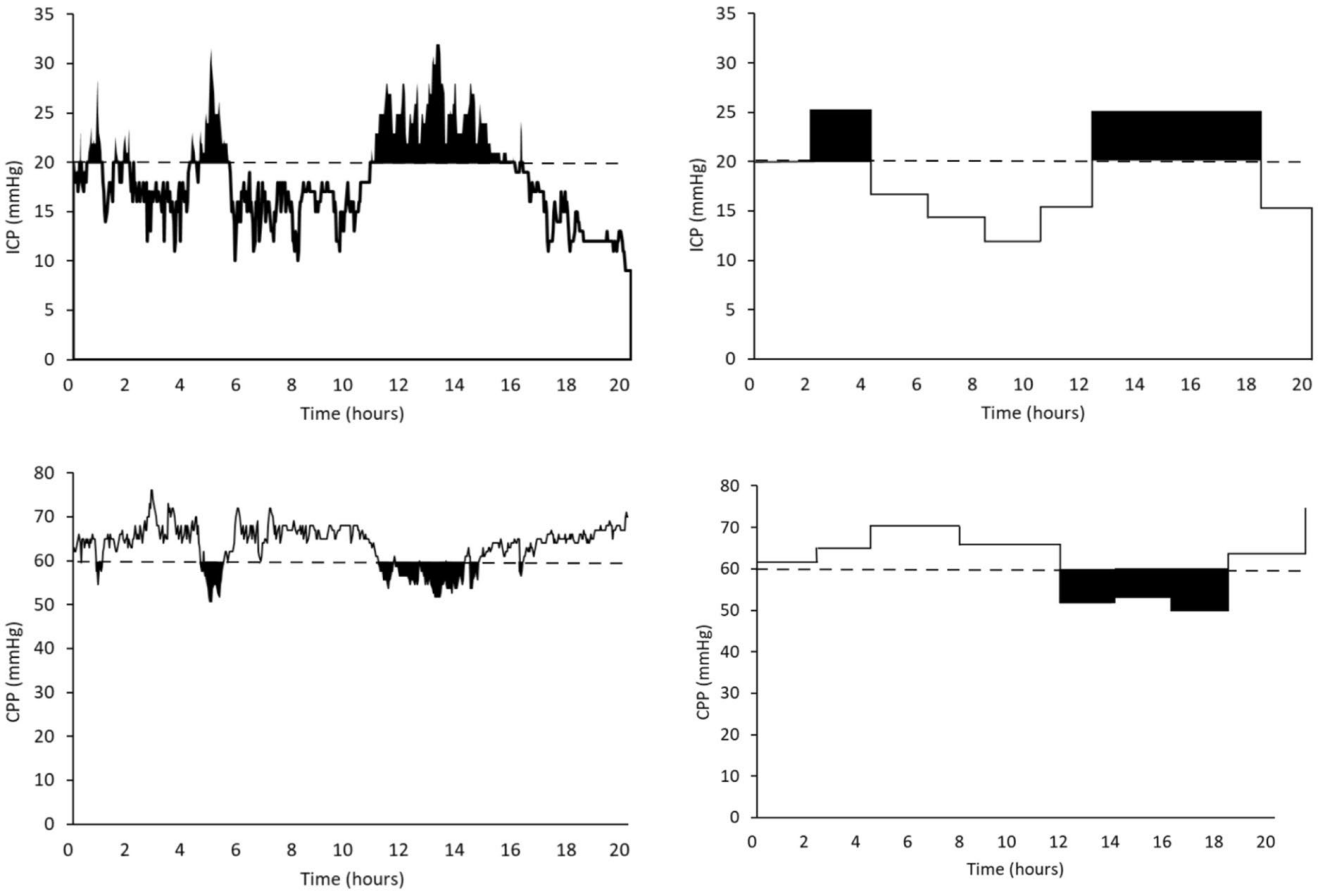
Calculation of intracranial pressure (ICP) pressure–time dose (PTD_ICP_) and cerebral perfusion pressure (CPP) pressure–time dose (PTD_CPP_). Upper left panel: the black area presents the PTD_ICP_ for the ICP threshold (ICP > 20 mm Hg, dashed line) from high-resolution (HR) data. Upper right panel: the black area presents the PTD_ICP_ for the ICP threshold (ICP > 20 mm Hg, dashed line) from the manually recorded (MR) data in the same patients. Because MR data were discontinuous, we used the staircase method for interpolation [19]. This assumes that an elevated ICP epoch begins at the first point at which an ICP > 20 mm Hg is recorded and ends when a new ICP is 20 mm Hg or lower. Lower left panels: the black area presents the PTD_CPP_ for the CPP threshold (CPP < 60 mm Hg, dashed line) from HR data. Lower right panel: the black area presents the PTD_CPP_ for the CPP threshold (CPP < 60 mm Hg, dashed line) from the MR data in the same patient, using the staircase method for interpolation [19]

The burden of intracranial hypertension and low CPP was calculated as the high (> 20 mm Hg) ICP pressure–time dose (PTD_ICP_) and the low (< 60 mm Hg) CPP pressure–time dose (PTD_CPP_) [20] (Fig. 2). PTD is an analytical method for computation of a cumulative dose of secondary injury by integrating the cumulative area under the curve above or below a defined physiological threshold. We use the entire monitoring time to calculate the PTD based on HR data and the staircase method to calculate the PTD based on MR data (Fig. 3). Patients were classified in three groups depending on the PTD [20]:

1. Low burden for patients with PTD less than 1 [PTD < 1 mm Hg/hour].
2. Medium burden for patients with PTD less than or equal to the median of the PTD > 1 distribution [PTD ≤ (median PTD > 1) mm Hg/hour].
3. High burden for patients with PTD greater than the median of the PTD > 1 distribution [PTD > (median PTD > 1) mm Hg/hour].

To assess whether MR ICP and CPP are representative of high ICP and low CPP burden we explored the relationship between burden groups, obtained from MR and HR data.

### Statistical Analysis

Data are presented as median and interquartile range (IQR). We used the Bland–Altman method to analyze the relationship between end-hour MR and HR values; HR values were considered the gold standard. Fisher’s exact test was used for contingency analysis.

## Results

### Patients’ Main Characteristics

One hundred patients (82 men) with a median age of 49 years (IQR 29–61) were studied. Their baseline characteristics are presented in Table 1. No significant differences were detected between the randomly included and excluded patients. The median Glasgow Coma Scale score was 6 (IQR 3–9), and only three patients had no visible pathological findings on the head CT scan. The median Glasgow Outcome Scale Extended score at 6 months was 4 (IQR 3–5). We analyzed 12,786 ICP and CPP monitoring hours (median per patient 124; IQR 101–164 h).

**Table 1:** Patients characteristics

Main characteristics of the patients	Included (*n* = 100)	Excluded (*n* = 53)
Age, median (IQR) yrs	49 (29–61)	49 (29–64)
Male, *n* (%)	82 (82)	40 (75)
Baseline GCS (after stabilization in the ED), median (IQR)	6 (3–9)	6 (3–8)
Baseline motor GCS, median (IQR)	4 (1–5)	4 (2–5)
Pupils baseline, one reacting (other nonreactive/missing/untestable), *n* (%)	9 (9)	6 (11)
Pupils baseline, both nonreactive, *n* (%)	16 (16)	9 (17)
Decompressive craniotomy, *n* (%)	23 (23)	9 (17)
GOSE at 6 months, median (IQR)	4 (3–5)	3 (3–5)
Marshall classification (admission imaging), n (%)		
1. No visible pathology on CT scan	3 (3)	3 (6)
2. Cisterns present, MLS < 5 mm	26 (26)	19 (35)
3. Cisterns compressed or absent, MLS < 5 mm	8 (8)	3 (6)
4. MLS > 5 mm, no mass lesion > 25 ml	1 (1)	1 (2)
5. Evacuated mass lesion	4 (4)	4 (8)
6. Nonevacuated mass lesion	46 (46)	17 (32)
No data on Marshall CT score	12 (12)	6 (11)

No statistically significant differences were detected between the two groups

*CT* computed tomography, *ED* emergency department, *GCS* Glasgow Coma Scale, *GOSE* Glasgow Outcome Scale Extended, *IQR* interquartile range, *MLS* mid-line shift

### End-Hour ICP and CPP

**Fig. 4:**
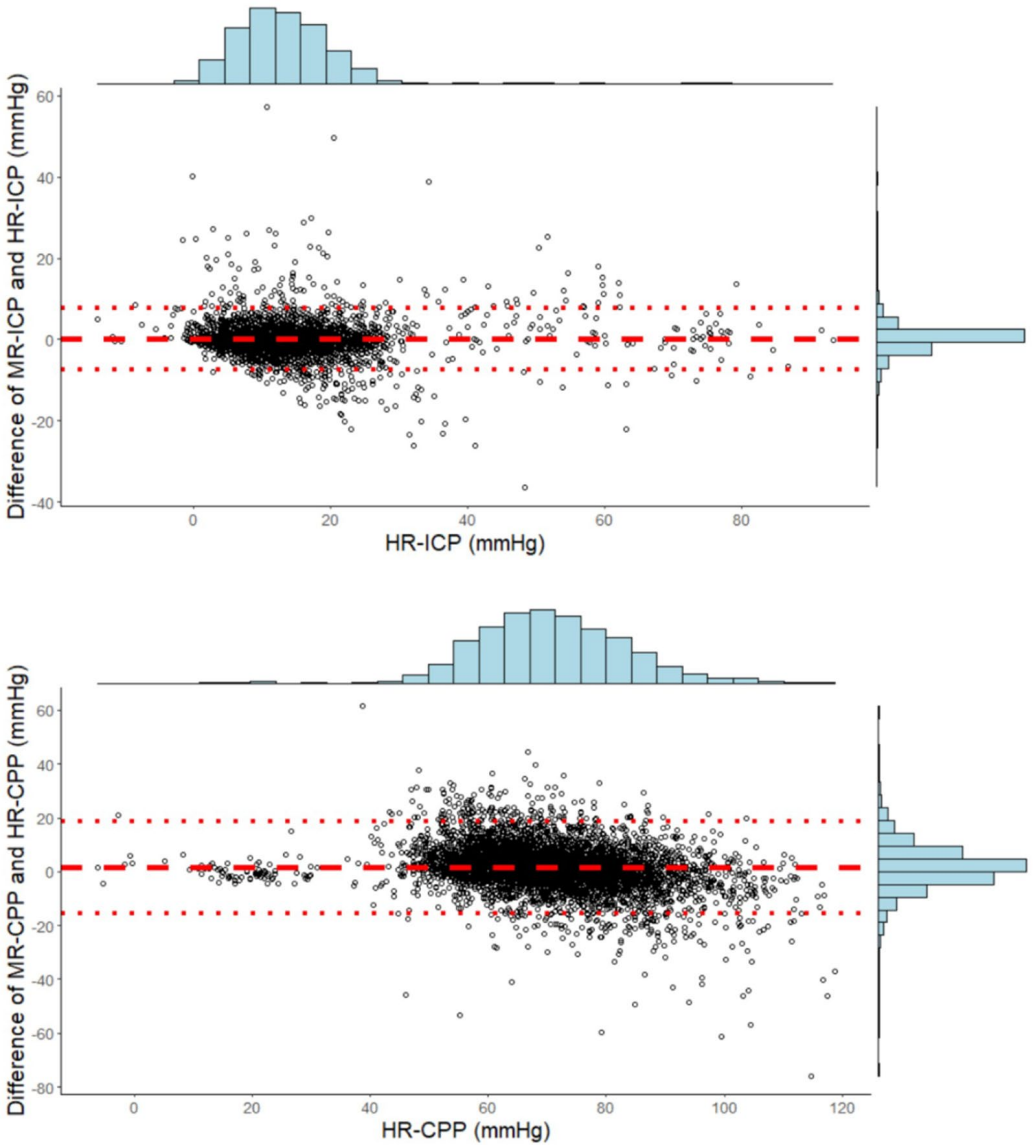
Bland–Altman plots comparing high-frequency and manual recording (MR) of end-hour intracranial pressure (ICP) and cerebral perfusion pressure (CPP). The mean difference between the two methods was 0.02 mm Hg for ICP (95% limits of agreement between − 7.54 and + 7.58 mm Hg) and 1.54 mm Hg for CPP (95% limits of agreement between − 15.72 and + 18.80 mm Hg)

We examined 6,393 two-hour intervals. There were 718 (11%) missing end-hour MR ICP values and 265 (4%) missing end-hour HR ICP values (*p* < 0.001). Therefore 5,484 synchronized simultaneous ICP values (median per patient 57; IQR 46–66) and 5,311 CPP values (median per patient 57; IQR 45–64) were available. Figure 4 graphically illustrates the Bland–Altman analysis: the mean difference between the two methods was 0.02 mm Hg for ICP (SD 3.86 mm Hg) and 1.54 mm Hg for CPP (SD 8.81 mm Hg). The limits of agreement, however, were fairly wide. The difference between HR and MR ICP was more than 5 mm Hg in 10% of comparisons (536 comparisons, median per patient 3; IQR 1–6), and MR ICP did not identify ICP > 20 mm Hg in 1.6% of comparisons. The difference between HR and MR CPP was more than 10 mm Hg in 16% of comparisons (896 comparisons, median per patient 6; IQR 3–13), and MR CPP did not detect CPP below 60 mm Hg in 5.8% of comparisons. Moreover, MR ICP was 20 mm Hg or less for 4,684 intervals, but HR detected episodes of intracranial hypertension in 21% of them. Similarly, MR CPP was 60 mm Hg or higher for 4,441 intervals, but episodes of low CPP were detected in 30% of them (Supplementary Fig. 1). Furthermore, we noted that in these intervals, the median percentage of time with ICP > 20 mm Hg was 18.3% (IQR 9–36) and the median percentage of time with CPP < 60 mm Hg was 21.5% (IQR 12–39).

### Pathological ICP and CPP Burden

The intensity and duration of high ICP and CPP were measured twice (see Methods for the PTD calculation). First, data stored in the computerized system were analyzed, and every single patient was classified as having a low, medium, or high burden of pathological ICP and CPP. The ICP burden was low in only four patients, medium in 42 patients, and high in 44 patients.

**Fig. 5:**
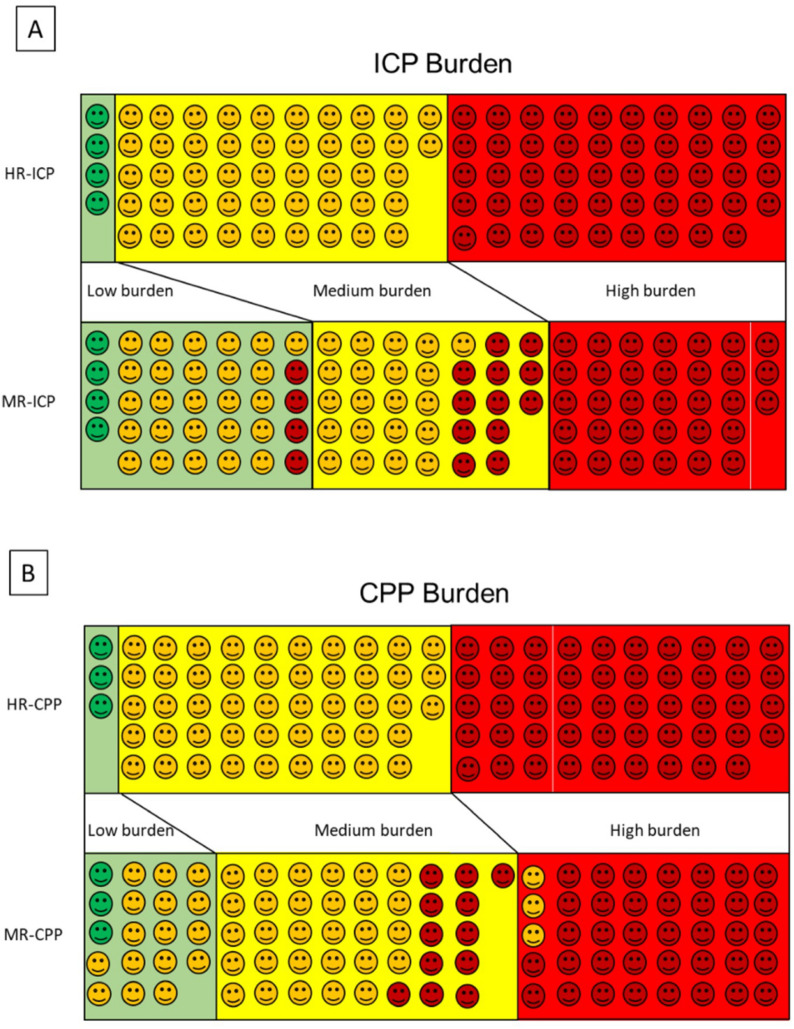
Graphic classification of high intracranial pressure (ICP) and low cerebral perfusion pressure (CPP) burden based on high-resolution (HR) recording and manual recording (MR) pressure–time dose (PTD). First, we classified the patients on the basis of HR PTD as low, medium, and high burden in the upper rows of panels **a** and **b**: low burden, green faces in green field; medium burden, yellow faces in yellow field; high burden, red faces in red field. Then we repeated the classification based on MR PTD in the lower columns of panel **a** and **b**: if a face is in a different field from the HR PTD classification, it indicates underestimation or overestimation of the burden. **a** Based on MR ICP, in 42 of 100 patients, the severity of high ICP is underestimated compared to the HR ICP classification. **b** Based on MR CPP, in 28 of 100 patients, the severity of low CPP is underestimated compared to HR CPP classification. In three patients, it was overestimated

Then a similar analysis was done on the manually recorded data, reclassifying each case in one of the three burden bands. The data indicated the ICP burden were low in 34 patients (including four patients previously classified as high burden), medium in 33 patients, and high in 33 patients. Figure 5 shows the results of this analysis with the two methods; differences in the bands are evident. A similar procedure was used for quantifying the CPP burden. In this analysis too, the classification based on manual entries underestimated the burden of pathological values.

## Discussion

The main goal of our study was to compare, in a large multicenter cohort of patients with TBI, the quality and quantity of information provided by manual or computerized ICP and CPP recording.

In most ICUs around the world, data provided by continuous electronic monitoring are manually annotated in the CRF by nurses and/or medical personnel. This does not require any particular dedicated technology or specific training and may take advantage of clinical expertise for filtering data (for instance, excluding artifacts, monitor disconnections, etc.). However, it relies on somewhat arbitrary choices, for instance, when a parameter changes rapidly and only a single value has to be selected or an average has to be estimated for the whole interval. An additional limitation is the risk of transcription errors. Finally, this task can only be done intermittently, usually every hour or so, whereas computerized recording offers continuous output at very fast sampling rates and unlimited storage with no additional work at the bedside; however, it can include artifacts and requires appropriate (often costly) technology with trained staff for inputting, storing, and filtering [16].

As part of the CENTER-TBI data recording, information on ICP and CPP were recorded manually every 2 h in 65 centers; only a subgroup of 21 centers also had ICP and CPP data simultaneously accumulated by a computerized system. The comparison of end-hour values entered in the CRF and the corresponding ones stored in the computerized system showed the data were very similar. On average, the data were consistent, with mean differences less than 2 mm Hg for both ICP and CPP. Average data, however, can be misleading because the MR values were higher in some instances and lower in other instances than the actual ICP or CPP. Fortunately, wide differences were rare (in the order of 10% for ICP differences greater than 5 mm Hg and 16% for CPP differences greater than 10 mm Hg). These results confirm that the staff in charge correctly entered the data provided by the monitoring equipment. This is in line with previous publications: our group in a single-center cohort of 30 patients with TBI showed good agreement between MR and digitally stored end-hour ICP (672 intervals) [18]. The BrainIT (Brain monitoring with Information Technology) collaborative network, in a multicenter study of 199 patients, also reported a good correlation between ICP and CPP from nursing charts (749 intervals) and computer-collected information [21].

The risk still exists that accurate but intermittent MR end-hour values may not fully document the perturbations of physiological signals in the clinical setting. Previous studies suggested that end-hour values were sufficient for ICP analysis in TBI [22, 23]. Because more recent studies have concentrated on the dose of intracranial hypertension or low CPP and its relationship with outcome [5–9, 24], we felt further investigation was warranted. PTD is a relatively simple method to summarize the burden of intracranial hypertension after TBI. This parameter seems to be associated with increased mortality and/or unfavorable outcome in several studies [7, 25–27]. Accurate measurement is therefore essential. Our findings indicate that the real burden of pathological ICP and CPP, measured by computerized methods, can be seriously underestimated if intermittent measurements are used. This is consistent with data from Hemphill and colleagues [19], who showed that PTD_ICP_ values strongly depend on the temporal resolution of data acquisition. In accordance with this, Kahraman et al. [26], in a study enrolling 30 study participants with TBI, found that PTD_ICP_ and PTD_CPP_ had no relation to long-term outcome if ICP and CPP were MR.

Our study has several limitations. First, we included in our analysis only 36% patients in the High-Resolution CENTER-TBI Sub-Study cohort. We excluded patients with external-ventricular-drain-based ICP monitoring for a practical reason: in these patients, ICP monitoring has often been interrupted for cerebrospinal fluid removal, and in large portions of the recording it, became difficult to identify a credible ICP measurement. As a consequence, our results could not be generalized to this subgroup of patients. Further, we included only patients with ICP monitoring lasting more than 72 h. Thus, we probably excluded patients in whom ICP was not pathological (leading to an early monitoring suspension). For this reason, it is possible that in this group of patients. the advantage of HR systems could be limited. Second, in the CENTER-TBI study the 2-h interval was chosen to reduce the workload in filling the CRF. We may speculate that shorter intervals (1 h or even 30 min) could improve the quantification of the ICP and CPP burden from MR data; on the contrary, the fair agreement between end-hour values should be confirmed. Last, we have restricted our analysis to the comparison of two current methods for data collection, not considering the relationship between ICP and outcome. This important topic was explored with proper statistical analysis in a recently published CENTER-TBI article [7] that included all the patients in the High-Resolution CENTER-TBI Sub-Study. Whether MR data could lead to different results could be the aim of further research.

## Conclusions

We demonstrate here the fair correspondence between end-hour ICP and CPP recorded manually and the same values recorded by a computer system. For accurate measurement of the burden of pathological values over time, however, a computerized system with continuous data collection does seem preferable.

## Supplementary Information

Below is the link to the electronic supplementary material.

Supplementary file1 (DOCX 118 kb)

## Data Availability

The data sets used in this study are available via https://www.center-tbi.eu/data on reasonable request.

## Abbreviations

TBI: Traumatic brain injury
ICP: Intracranial pressure
CPP: Cerebral perfusion pressure
ICU: Intensive care unit
CRF: Case report form
MR-ICP and MR-CPP: Manually recorded end-hour ICP and CPP
HR-ICP and HR-CPP: ICP and CPP recorded by a computerized high-resolution system
GOSE: Glasgow outcome scale extended
EVD: External ventricular drain
CSF: Cerebrospinal fluid
CT: Computed tomography
SBP: Systolic blood pressure
DBP: Diastolic blood pressure
MAP: Mean arterial blood pressure
HR: High resolution
PTD: Pressure time dose
AUC: Area under curve
GCS: Glasgow coma scale
IQR: Interquartile range
