# Correction: Accuracy of Manual Intracranial Pressure Recording Compared to a Computerized High-Resolution System: A CENTER-TBI Analysis

**DOI:** 10.1007/s12028-023-01722-4

**Published:** 2023-04-14

**Authors:** Tommaso Zoerle, Tatiana Birg, Marco Carbonara, Peter Smielewski, Michal M. Placek, Elisa R. Zanier, Cecilia A. I. Åkerlund, Fabrizio Ortolano, Nino Stocchetti

**Affiliations:** 1 Neuroscience Intensive Care Unit, Department of Anesthesia and Critical Care, Fondazione IRCCS Ca’ Granda Ospedale Maggiore Policlinico, Milan, Italy; 2 Department of Pathophysiology and Transplantation, University of Milan, Milan, Italy; 3 Brain Physics Lab, Division of Neurosurgery, Department of Clinical Neurosciences, Addenbrooke’s Hospital, University of Cambridge, Cambridge, UK; 4 Department of Neuroscience, Istituto Di Ricerche Farmacologiche Mario Negri IRCCS, Milan, Italy; 5 Department of Physiology and Pharmacology, Section of Perioperative Medicine and Intensive Care, Karolinska Institutet, Stockholm, Sweden

**Correction: Neurocrit Care** https://doi.org/10.1007/s12028-023-01697-2

The original article has been updated to include the author names and affiliations of the CENTER-TBI High Resolution ICU Sub-Study Participants and Investigators in the Acknowledgements section.

