# Supplementary material for: Accuracy of Manual Intracranial Pressure Recording Compared to a Computerized High-Resolution System: A CENTER-TBI Analysis
Source: Neurocrit Care. 2023 Mar 15;38(3):781–90. doi: 10.1007/s12028-023-01697-2 (PMC10241732; doi:10.1007/s12028-023-01697-2)

**Additional file 1:** MR-ICP <20 mmHg and MR-CPP >60 mmHg and high ICP/low CPP episodes in the corresponding two-hour HR interval


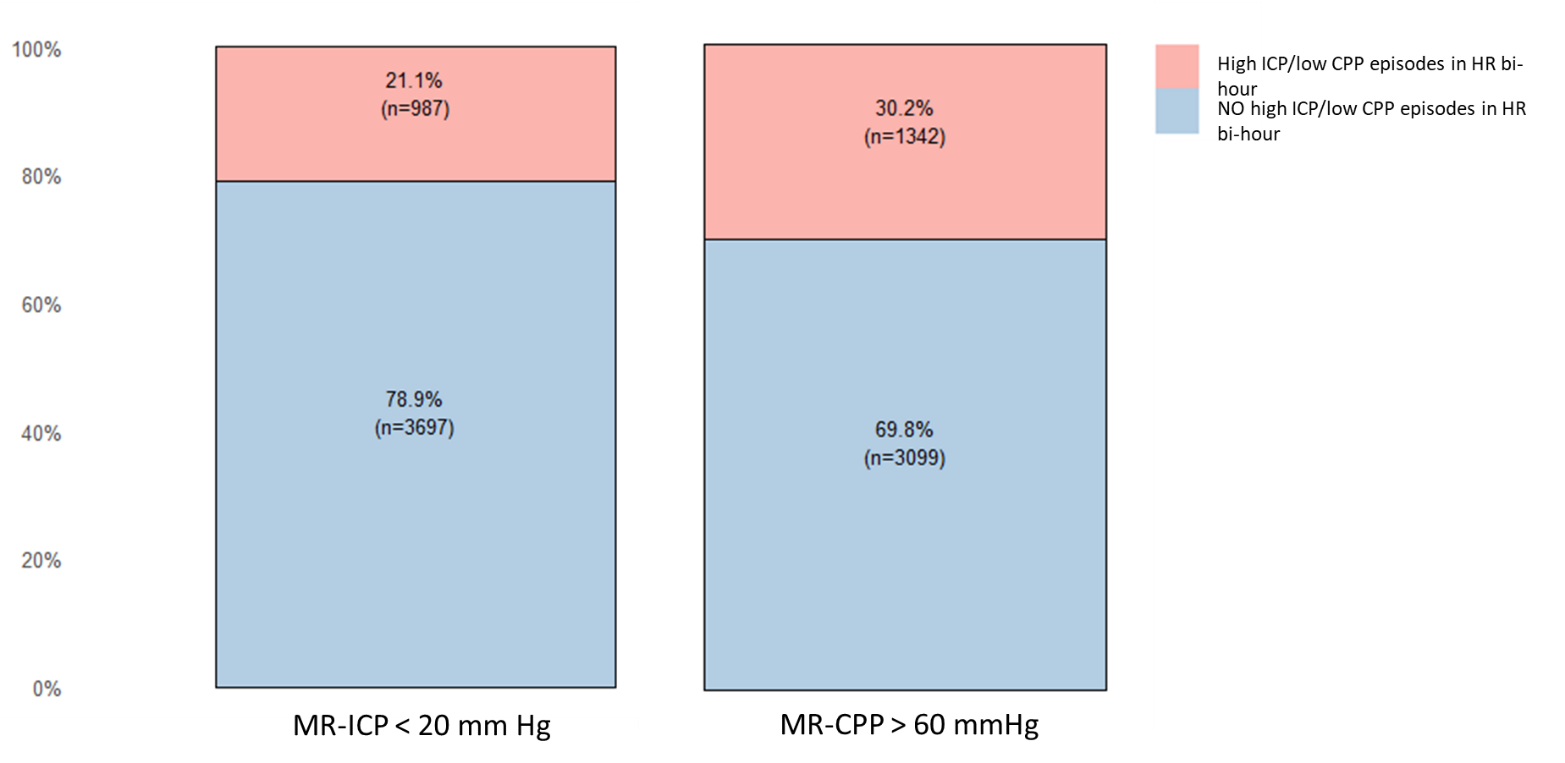

Supplement: Supplementary file 1 — Supplementary file1 (DOCX 118 kb) [file 12028_2023_1697_MOESM1_ESM.docx]
